# Distinguishing West Nile virus infection using a recombinant envelope protein with mutations in the conserved fusion-loop

**DOI:** 10.1186/1471-2334-14-246

**Published:** 2014-05-09

**Authors:** Stefan Chabierski, Luisa Barzon, Anna Papa, Matthias Niedrig, Jonathan L Bramson, Justin M Richner, Giorgio Palù, Michael S Diamond, Sebastian Ulbert

**Affiliations:** 1 Department of Immunology, Fraunhofer Institute for Cell Therapy and Immunology, Perlickstrasse 1, 04103 Leipzig, Germany; 2 Department of Molecular Medicine, University of Padova, via Gabelli 63, 35121 Padova, Italy; 3 Department of Microbiology, Medical School, Aristotle University of Thessaloniki, 54124 Thessaloniki, Greece; 4 Robert Koch Institute, 13353 Berlin, Germany; 5 McMaster Immunology Research Centre, McMaster University, Hamilton, Ontario L8N 3Z5, Canada; 6 Departments of Medicine, Molecular Microbiology, Pathology & Immunology, Washington University School of Medicine, 63110 St Louis, MO, USA

**Keywords:** West Nile virus, Diagnosis, Antibodies, Envelope protein

## Abstract

**Background:**

West Nile Virus (WNV) is an emerging mosquito-transmitted flavivirus that continues to spread and cause disease throughout several parts of the world, including Europe and the Americas. Specific diagnosis of WNV infections using current serological testing is complicated by the high degree of cross-reactivity between antibodies against other clinically relevant flaviviruses, including dengue, tick-borne encephalitis (TBEV), Japanese encephalitis (JEV), and yellow fever (YFV) viruses. Cross-reactivity is particularly problematic in areas where different flaviviruses co-circulate or in populations that have been immunized with vaccines against TBEV, JEV, or YFV. The majority of cross-reactive antibodies against the immunodominant flavivirus envelope (E) protein target a conserved epitope in the fusion loop at the distal end of domain II.

**Methods:**

We tested a loss-of-function bacterially expressed recombinant WNV E protein containing mutations in the fusion loop and an adjacent loop domain as a possible diagnostic reagent. By comparing the binding of sera from humans infected with WNV or other flaviviruses to the wild type and the mutant E proteins, we analyzed the potential of this technology to specifically detect WNV antibodies.

**Results:**

Using this system, we could reliably determine WNV infections. Antibodies from WNV-infected individuals bound equally well to the wild type and the mutant protein. In contrast, sera from persons infected with other flaviviruses showed significantly decreased binding to the mutant protein. By calculating the mean differences between antibody signals detected using the wild type and the mutant proteins, a value could be assigned for each of the flaviviruses, which distinguished their pattern of reactivity.

**Conclusions:**

Recombinant mutant E proteins can be used to discriminate infections with WNV from those with other flaviviruses. The data have important implications for the development of improved, specific serological assays for the detection of WNV antibodies in regions where other flaviviruses co-circulate or in populations that are immunized with other flavivirus vaccines.

## Background

The mosquito-transmitted West Nile Virus (WNV) belongs to the *Flaviviridae* family of positive stranded RNA viruses, which also includes other arthropod-borne viruses such as dengue (DENV), tick borne encephalitis (TBEV), Japanese encephalitis (JEV), and yellow fever (YFV) viruses. WNV circulates in nature between mosquitoes and birds, but humans and other mammals also can be infected. In humans, about twenty percent of infected individuals develop flu-like symptoms, whereas in a subset of patients, primarily the elderly and immunocompromised, severe and sometimes fatal neurological complications can develop [1]. WNV was first isolated in Africa and later found to circulate in Asia, Australia, and sporadically in Europe. WNV was introduced into the United States in 1999 and rapidly spread throughout the Americas in the ensuing decade [2]. In addition, WNV has become endemic in several Southern and Eastern European countries during the past five years [3-6].

Several genetic lineages of WNV exist, and most isolates belong either to lineage 1 or lineage 2. Whereas in the Americas only WNV strains belonging to lineage 1 have been identified, in Europe strains of lineages 1 and 2 are circulating, sometimes even in the same area [7,8].

WNV infections can be diagnosed by directly detecting the viral RNA, or by measuring antibodies produced against it in serum or cerebrospinal fluid (CSF). As viremia is transient, of low magnitude, and often precedes clinical manifestations, RNA detection can be challenging. In comparison, IgM antibodies are produced approximately 4 to 7 days after infection and IgG antibodies appear a few days later [9]. Therefore, antibody-based detection systems, such as ELISAs or indirect immunofluorescence tests, are commonly used for WNV diagnosis. However, a limitation of serological diagnosis for WNV infection is the structural similarity of the immunodominant envelope (E) protein among Flavivirus genus members. Antibodies produced against the E protein can be cross-reactive, leading to false-positive test results [10-12]. This problem occurs in many parts of the world due to co-circulation of different flaviviruses and historical vaccination with live attenuated or inactivated TBEV, JEV, or YFV vaccines. In Europe, cross-reactivity of antibodies against TBEV and WNV has been observed, especially in countries where TBEV vaccination is common [13]. Consequently, positive results obtained with the existing methods must be confirmed by lower-throughput virus neutralization tests, which require high-security and biosafety laboratories, which adds to the expense of the testing and delay in establishing a diagnosis [14].

Previous work has established that cross-reactive antibodies target the highly conserved fusion loop of the flavivirus E protein [15]. Moreover, binding of such cross-reactive antibodies can be diminished by inserting mutations into this epitope in the E protein or in virus-like particles (VLPs) [16-20]. Here, using bacterially expressed wild type or loss-of-function mutant WNV E proteins, we evaluated the binding of antisera derived from humans infected with different flaviviruses. This assay allowed us to determine rapidly and reliably WNV infections.

## Methods

### Antigens

The WNV E ectodomain (amino acid residues 1 to 404) and the quadruple mutant (T76A, M77G, W101R, L107R) of the New York 1999 strain (Acc. Nr. FJ151394) were expressed from the pET21a plasmid in *Escherichia coli*, and purified after an oxidative refolding protocol, as described previously [20,21]. The proteins were isolated as a monodispersed peak on a Superdex 75 or 200, 16/60 size-exclusion column using fast-protein liquid chromatography (GE Healthcare).

### Serum samples

Serum samples from confirmed WNV-infections (described in [22]) were obtained during outbreaks in Italy and Greece in 2010. The Italian samples (University of Padova, Italy) were derived from seroprevalence studies, blood donors or patients with West Nile neuroinvasive disease. Ethical approval was obtained from the Padova University Hospital ethics committee. The Greek samples (University of Thessaloniki, Greece) were obtained from patients with neuroinvasive disease, taken during the acute phase of illness (3–17 days). WNV infections were confirmed by virus neutralization tests. Ethical approval was obtained from the Medical School of Aristotle University of Thessaloniki ethics committee. Two WNV-positive samples were obtained from Seracare Life Sciences (USA). Serum samples from Canada were obtained from patients with confirmed WNV-specific T-cell responses [21]. The study was approved by the Hamilton Health Sciences/McMaster Health Sciences research ethics board. None of the patients was vaccinated against other flaviviruses or had a recent travel history to other countries endemic for WNV. Serum samples from JEV-vaccinated individuals participating in a randomized controlled vaccination study (approved by the national ethics committee) were obtained from the Robert-Koch Institute (Berlin, Germany). Sera from confirmed TBEV and DENV-infected individuals and negative controls were obtained from Padova University Hospital (Italy). All confirmed DENV cases were international travellers returning from endemic countries with diagnosis of recent primary DENV infection and with laboratory tests positive for IgM/IgG or IgG against only DENV. Confirmed TBEV IgG-positive serum samples were selected from a seroprevalence study in forest rangers. The TBEV IgG-positive samples were from subjects vaccinated against TBEV or with a history of confirmed TBEV infection. The neutralizing titer for WNV was negative in all of these cases (data not shown). Ethical approval was obtained from the Padova University Hospital ethics committee.

All persons participating in this research provided informed consent and all samples were analyzed anonymously.

Antibodies against DENV were detected by using DENV IgG and IgM capture DxSelect (Focus Diagnostics, Cypress, CA, USA). Antibodies against TBEV were tested by using anti-TBE Virus IgG, IgM Enzygnost® ELISA (Siemens Healthcare, Germany).

### Antibody measurements

Nunc polysorb plates (Thermo Scientific, Germany) were coated overnight with indicated amounts of recombinant E ectodomain protein or E-quadruple mutant (in coating buffer (15 mM Na_2_CO_3_, 35 mM NaHCO_3_ pH 9.6)) per well with gentle agitation at 4°C. The plates were washed three times with 350 μL per well of PBS/Tween (0.05%), followed by blocking with 5% non-fat dry milk powder (200 μL per well) for 2 h at room temperature (RT). After a second wash step, human sera (dilution 1:100 in 5% non-fat dry milk powder, 100 μL per well) were incubated for 1.5 h at RT. The sera were removed by a third wash step and 100 μL of the secondary antibody (1:10.000 diluted HRP-conjugated Goat-anti-Human IgG (Fisher Scientific)) was added for 1 h at RT. After washing, the TMB-substrate (BioLegend, Germany) was added to the wells and the plate was incubated for 30 min at RT in darkness. To stop the reaction, 1 M H_2_SO_4_ was added, followed by measurement at 450 nm and 520 nm (reference wavelength) in an ELISA Reader (Infiniti M200, Tecan). All antibody tests were performed in duplicates in at least two independent experiments.

Equal loading of wild type and mutant E protein was verified using the humanized E16 monoclonal antibody (dilution 1:1000), which targets an epitope on domain III of the E protein, distant from the fusion loop [23] (data not shown).

### Statistical analysis

Statistical analysis was performed using Mann–Whitney Rank Sum Test in SigmaStat.

## Results and discussion

**Figure 1:**
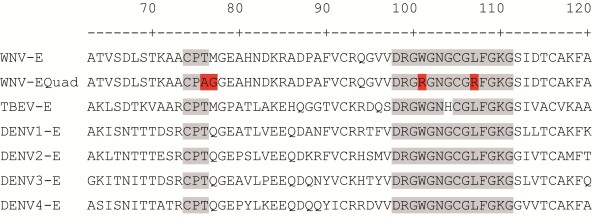
**Sequence alignment of fusion loop domain of E proteins from different flaviviruses, WNV E: West Nile virus wild type E-protein, WNV-Equad: WNV E-quadruple mutant (point mutations are marked in red), TBEV-E: TBEV, DENV: DENV E protein, serotypes 1–4.** Amino acids positions of the full length protein are indicated in the top row.

**Figure 2:**
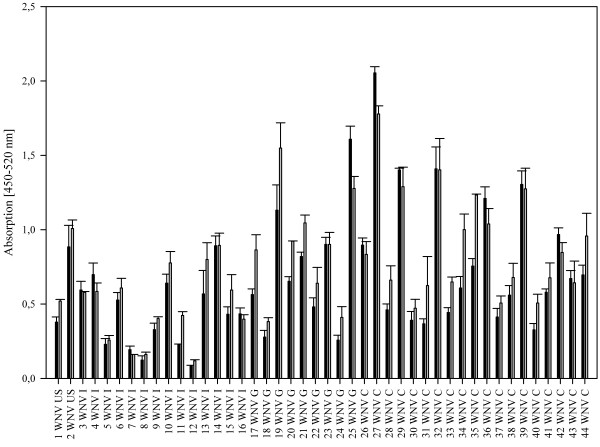
Analyses of WNV infected human sera on 50 ng per well of the microtiter plate of E wild type (grey columns) compared to the mutant Equad (black columns), sera from different areas, as indicated next to the numbers (G: Greece, I: Italy, C: Canada, US: USA).

**Figure 3:**
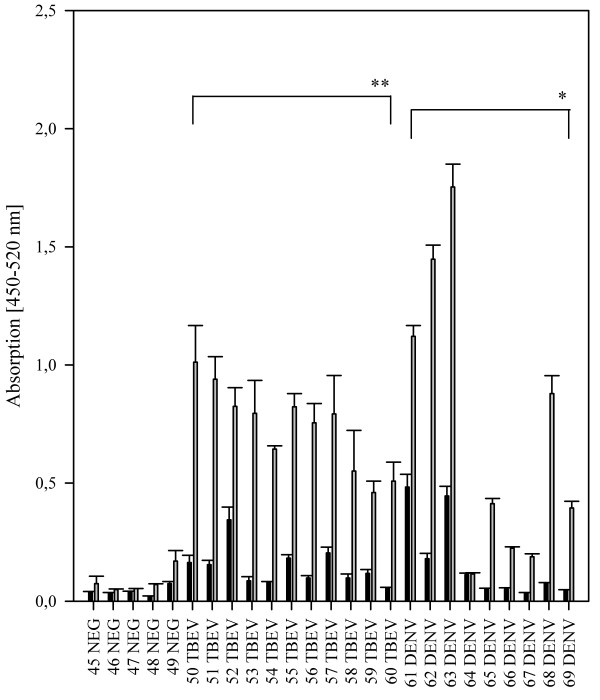
**Comparison of IgG binding to 50 ng per well of wild type E (grey columns) and Equad (black columns) using negative (NEG), TBEV- or DENV- positive sera.** Asterisks indicate statistically significant differences between wild type and mutant WNV-Envelope protein (TBEV: **, *P* = <0.001; DENV: *, *P* = 0.010; Mann–Whitney Rank Sum Test).

To analyze the influence of the E protein fusion loop on the specificity of flavivirus IgG antibody binding to the E protein, we used a bacterially expressed wild type E-protein and a loss-of-function mutant (Equad), which contains four mutations within and proximal to the fusion loop [20] (Figure 1). Both proteins were incubated with sera from humans infected with WNV from outbreaks in Europe and America. Although the overall signal strength varied substantially between individuals, all samples showed clearly detectable signals that did not differ markedly between the wild type and the mutant E-proteins (Figure 2). Binding was similar in sera from patients infected with WNV strains belonging to genetic lineage 1 or lineage 2 [24], as demonstrated by signals obtained with sera from the United States, Canada or Italy (lineage 1) and Greece (lineage 2). Next, the assay was evaluated for its specificity by testing sera from humans containing antibodies against the heterologous flaviviruses, TBEV and DENV. In contrast to the WNV-positive samples, we observed statistically significant differences in binding between the wild-type and quadruple mutant E proteins (Figure 3). Most samples showed the expected strong cross-reactivity, with high binding to wild type WNV E; however, the values for binding of the heterologous sera to the fusion loop mutant were substantially lower. Although some DENV-infected sera showed binding to the mutant WNV E protein, the values obtained with the wild type E protein were all significantly higher.

**Figure 4:**
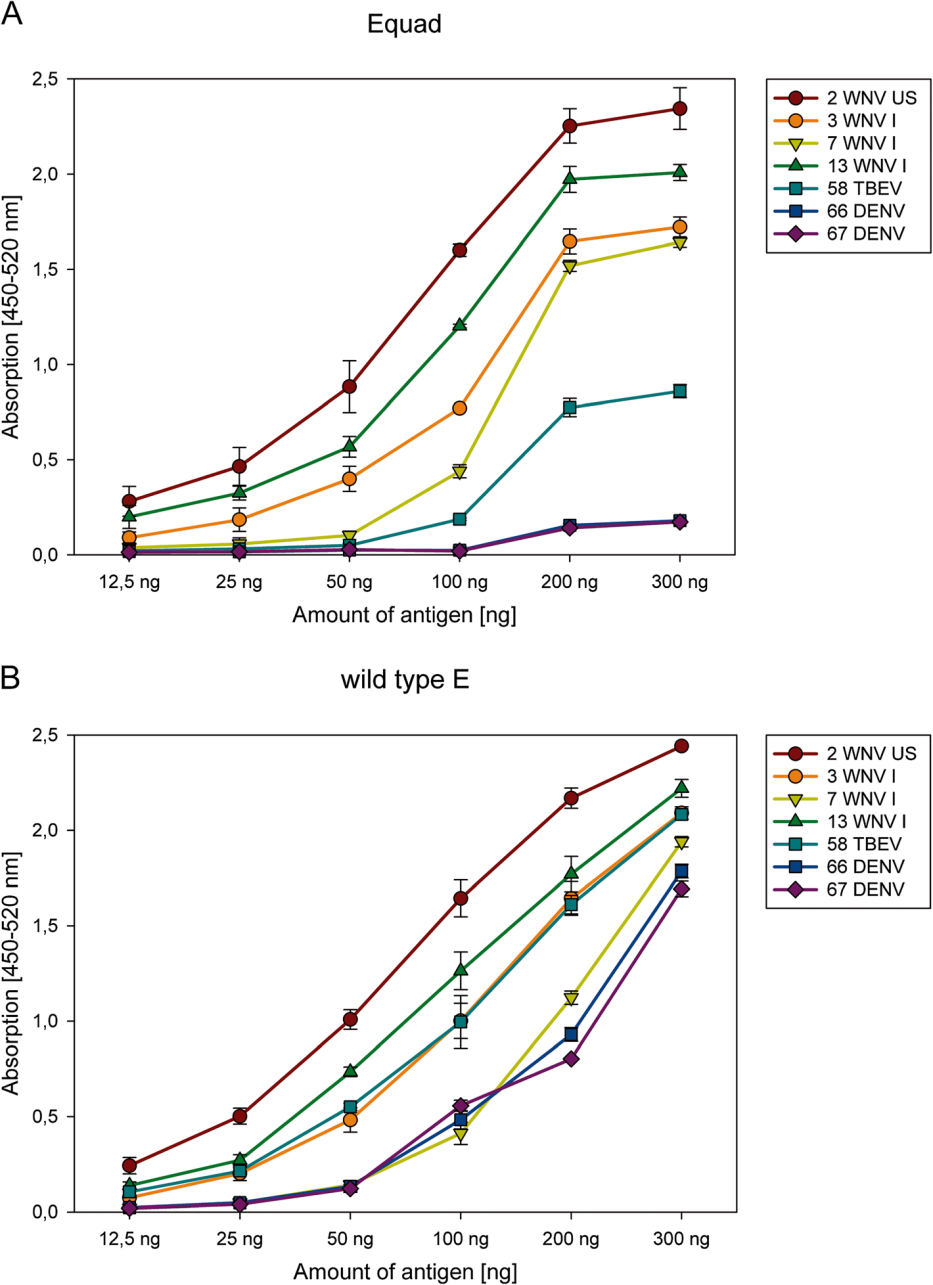
**Antigen titration of Equad (A) and E wild type (B).** Different sera were incubated with an increasing amount of recombinant proteins per well of the microtiter plate. Differences between individual sera at 300 ng of antigen were not statistically significant.

**Figure 5:**
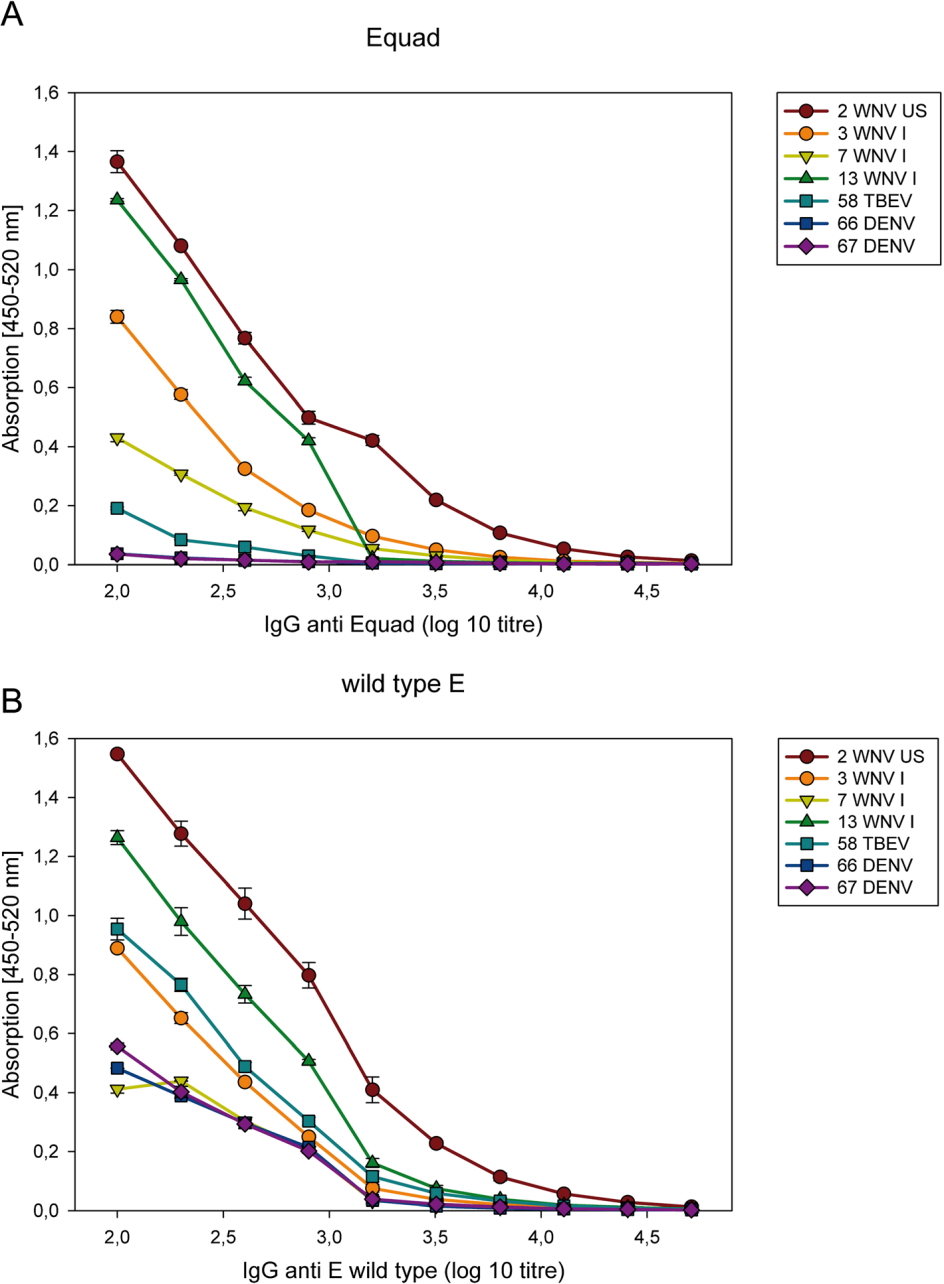
Sera dilution curves using constant antigen amounts (100 ng) of the Equad mutant (A) and wild type E protein (B).

To assess the relative amount of antibody against wild type and mutant E proteins, sera from WNV-, TBEV- or DENV-infected individuals were incubated with increasing amounts of the two protein antigens in the solid phase. For the WNV-positive sera, the signal for binding the Equad mutant saturated at ~200 ng per well (Figure 4). In contrast, saturation was not observed with the wild type protein until 300 ng of protein was added. This possibly reflects the limiting amount of WNV-specific antibodies that target other non-fusion loop epitopes. Increasing the antigen amount from 50 ng to 100 ng resulted in enhanced detection of sera, especially for those showing moderate binding at the lower amounts, as exemplified by WNV serum 7 (see Figure 2), but increased the background binding of some samples (data not shown). Serum dilution assays confirmed the marked differences in titers against the mutant and the wild type E protein shown by the DENV- and TBEV positive sera (Figure 5).

By calculating the average ratios between the signal for the wild-type and mutant protein in Figures 1 and 2, a value could be assigned for each of the flaviviruses, which distinguished their pattern of reactivity. For WNV, the average ratio was 1.22 (standard deviation (SD) of 0.2), for DENV 5.92 (SD of 3.1) and for TBEV 6.06 (SD of 2.1), with a statistically significant difference between WNV and the other two infections (*P* = 0.005, Mann–Whitney Rank Sum Test).

**Figure 6:**
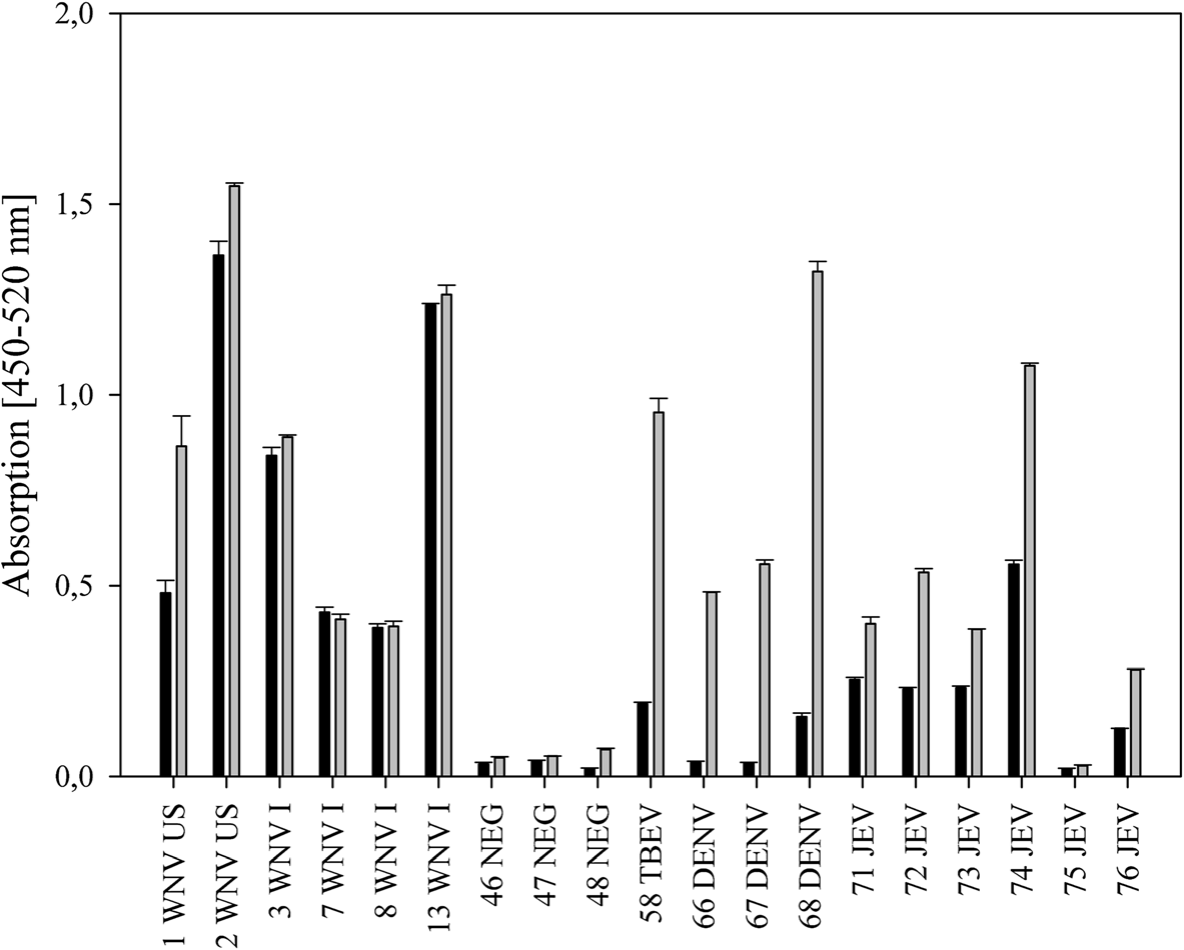
Comparison of IgG binding to wild type E (grey columns) and Equad (black columns) using negative (NEG), WNV-, TBEV-, DENV- and JEV-positive sera.

To analyze the suitability of this system to discriminate between infections with different flaviviruses of the JEV-serocomplex, we analyzed sera from individuals vaccinated against JEV. Due to the low antibody titres in these sera 100 ng of antigen per well were required. Similar to the DENV and TBEV infections, in all samples there was decreased binding to the Equad antigen compared to the wild-type E protein (Figure 6). However, under the conditions used, the differences were less pronounced and not statistically significant when compared to the WNV samples, a finding which is not unexpected given the higher amino acid sequence identity (approx. 80%) of the WNV E-protein to JEV as compared to TBEV or DENV (approx. 40% and 50%, respectively). This indicates that the principle of discrimination also applies to JEV, but the definition of average ratios will require more refinement for WNV-related viruses from the same JEV serocomplex.

In summary, we present a new assay for the serologic diagnosis of WNV infections, which is based on the relative difference in antibody binding to mutant and wild type E protein of WNV. The data suggest that defined values could be established that allow the differentiation of flavivirus infections. The observation that antibody binding to the E protein varied substantially among WNV-infected individuals is consistent with previous observations describing the heterogeneity of the human humoral immune response to WNV infections [22,25]. However, for WNV, there were no significant differences observed between the binding towards wild type and mutant E protein, unless the antigens were present at high density. Because the human antibody response against WNV is skewed towards non-neutralizing epitopes including the fusion loop [21,26], some difference in binding was expected. However, DENV- and TBEV- positive sera show a more pronounced difference in the binding to wild type and mutant E protein, which reflects the immunodominance of the fusion-loop epitope as a cross-reactive determinant [18,27-29]. Although diminished binding of cross-reactive DENV-infected sera using a similar Equad protein has been shown previously [20], sera from WNV- and TBEV infected patients were not analyzed in that study. The quadruple mutant contains four mutations adjacent to and within the fusion loop of the protein, which impact the binding of cross-reactive flavivirus antibodies. Using a VLP-based system, Roberson et al. [19] analyzed a double-mutant (G106R and L107H) in the fusion loop of the E protein for WNV diagnosis. Our approach differs in number and positions of the mutations and in the antigen platform. A recombinant bacteria-derived protein has the advantage over mammalian cell-culture derived VLPs as it can be produced rapidly, inexpensively, and in higher yield, and likely can be quantified more precisely for diagnostic applications. Therefore, our results may be useful for the development of a specific rapid diagnostic test for the detection of WNV IgG and possibly, IgM antibodies. IgM detection would be particularly useful for the investigation of recent infections. Alternatively, a recent infection can also be diagnosed by measuring a rise in IgG antibody titers over time [11].

## Conclusions

By using a recombinant loss-of-function mutant of the WNV E-protein, infections with WNV can be discriminated from those with TBEV and DENV based on antibody measurements. Whereas under the conditions used no substantive difference in binding of WNV antibodies to the wild type or mutant E protein was observed, anti-TBEV or –DENV antibodies bound significantly less well to the mutant protein lacking the cross-reactive fusion loop epitope.

## Competing interests

The authors declare that they have no competing interest.

## Authors’ contributions

SC and JMR carried out the immunoassays and produced the recombinant antigens. SU, SC and MSD conceived and designed the experiments and wrote the paper. LB, MN, AP, JLB and GP contributed reagents/materials/analysis tools and added important intellectual input into study design and writing of the manuscript. All authors read and approved the final manuscript.

## Pre-publication history

The pre-publication history for this paper can be accessed here:

http://www.biomedcentral.com/1471-2334/14/246/prepub
